# Characterizing Canadian long-term care home consumed foods and their inflammatory potential: a secondary analysis

**DOI:** 10.1186/s12889-022-14934-8

**Published:** 2023-02-06

**Authors:** Kaylen J. Pfisterer, Robert Amelard, Heather H. Keller, Alexander Wong

**Affiliations:** 1 Department of Systems Design Engineering, University of Waterloo, 200 University Ave W, N2L 3G1 Waterloo, Canada; 2 Waterloo Artificial Intelligence Institute, 200 University Ave W, N2L 3G1 Waterloo, Canada; 3 Schlegel-University of Waterloo Research Institute for Aging, 250 Laurelwood Drive, N2J 0E2 Waterloo, Canada; 4 Centre for Digital Therapeutics, University Health Network, 550 University Ave, M5G 2A2 Toronto, Canada; 5 KITE-Toronto Rehabilitation Institute, University Health Network, 550 University Ave, M5G 2A2 Toronto, Canada; 6 Department of Kinesiology and Health Studies, University of Waterloo, 200 University Ave W, N2L 3G1 Waterloo, Canada

**Keywords:** Long-term care, Food intake patterns, Canada’s Food Guide, Dietary Inflammatory Index, Dietary intake, Aging, Artificial intelligence

## Abstract

**Background:**

Nutrient dense food that supports health is a goal of food service in long-term care (LTC). The objective of this work was to characterize the “healthfulness” of foods in Canadian LTC and inflammatory potential of the LTC diet and how this varied by key covariates. Here, we define foods to have higher “healthfulness” if the are in accordance with the evidence-based 2019 Canada’s Food Guide, or with comparatively lower inflammatory potential.

**Methods:**

We conducted a secondary analysis of the Making the Most of Mealtimes dataset (32 LTC homes; four provinces). A novel computational algorithm categorized food items from 3-day weighed food records into 68 expert-informed categories and Canada’s Food Guide (CFG) food groups. The dietary inflammatory potential of these food sources was assessed using the Dietary Inflammatory Index (DII). Comparisons were made by sex, diet texture, and nutritional status.

**Results:**

Consumption patterns using expert-informed categories indicated no single protein or vegetable source was among the top 5 most commonly consumed foods. In terms of CFG’s groups, protein food sources (i.e., foods with a high protein content) represented the highest proportion of daily calorie intake (33.4%; animal-based: 31.6%, plant-based: 1.8%), followed by other foods (31.3%) including juice (9.8%), grains (25.0%; refined: 15.0%, whole: 10.0%), and vegetables/fruits (10.3%; plain: 4.9%, with additions: 5.4%). The overall DII score (mean, IQR) was positive (0.93, 0.23 to 1.75) indicating foods consumed tend towards a pro-inflammatory response. DII was significantly associated with sex (female higher; p<0.0001), and diet (minced higher; p=0.036).

**Conclusions:**

“Healthfulness” of Canadian LTC menus may be enhanced by lowering inflammatory potential to support chronic disease management through further shifts from refined to whole grains, incorporating more plant-based proteins, and moving towards serving plain vegetables and fruits. However, there are multiple layers of complexities to consider when optimising foods aligned with the CFG, and shifting to foods with anti-inflammatory potential for enhanced health benefits, while balancing nutrition and ensuring sufficient food and fluid intake to prevent or treat malnutrition.

**Supplementary Information:**

The online version contains supplementary material available at 10.1186/s12889-022-14934-8.

## Background

Nutrition plays a crucial role in overall health and well-being. Yet, long-term care resident malnutrition (or risk) prevalence is high (54% [1] versus 19% to 42% based on 37 studies, 17 countries [2]). As part of monitoring nutritional status in LTC, understanding what foods and fluids are most commonly consumed and their “healthfulness” may provide insight into how foods and fluids provided may be managed to support health (e.g., [3–7]) and inform tailored recommendations to reduce disease burden.

The 2019 Canada’s Food Guide (CFG) revision provides recommendations to support optimal health based on current available evidence [8]. Although not specifically designed for a LTC population, a nutrient dense diet is still a goal for LTC where, “optimal health” may be considered as supporting chronic disease management, delaying or slowing the progression of conditions, and seeking to increase quality of life while balancing flexibility and emphasizing enjoyment to support a food-first approach to nutritional adequacy. For example, food sources which place an emphasis on consuming more vegetables, fruits, and whole grains imparts a reduced risk of cardiovascular disease and lowers blood lipids, and reduced salt intake reduces high blood pressure [9]. One mechanism of action for a healthy diet as ascribed by CFG may be through the role of inflammatory potential of foods. Not only can more plant-based whole foods (i.e., higher vegetable, fruit, and whole grain intake) decrease chronic inflammation, they may reduce the risk of cardiovascular disease, and protect against frailty [10]. Within LTC there is substantial chronic disease burden; in Ontario LTC 90% of LTC residents have cognitive impairment, 76% have cardiovascular diseases, and 62% have musculoskeletal diseases like arthritis or osteoporosis [11]. There is also strong evidence to support the role of inflammation in many of these pervasive chronic diseases including: diabetes [12]; cognitive impairment and dementia [13–19]; cardiovascular disease, heart failure, and hypertension [20–25]; and musculoskeletal diseases including arthritis and osteoporosis [26–29]). Taken together, this suggests there may be additional value in characterizing food sources pertaining to inflammation beyond CFG particularly as an opportunity to support chronic disease management through reducing inflammatory potential of consumed foods.

Beyond nutrient adequacy, the “healthfulness” of the diet has not been determined and comparison to CFG and DII provides an opportunity to examine a longstanding concern of residents and families - that is, is the food provided in LTC as healthy as it can be? The purpose of this secondary analysis of representative Canadian LTC food consumption data (Making the Most of Mealtimes (M3)) is to address three research questions: (1) How can Canadian LTC residents’ food and fluid consumption be characterized with respect to the 2019 CFG food groups?; (2) What is the “inflammatory potential” of these foods and fluids?; and (3) How does inflammatory potential of these consumed foods vary by the key variables: sex, diet texture (e.g., minced, puréed), and nutritional status?

## Methods

**Fig. 1:**
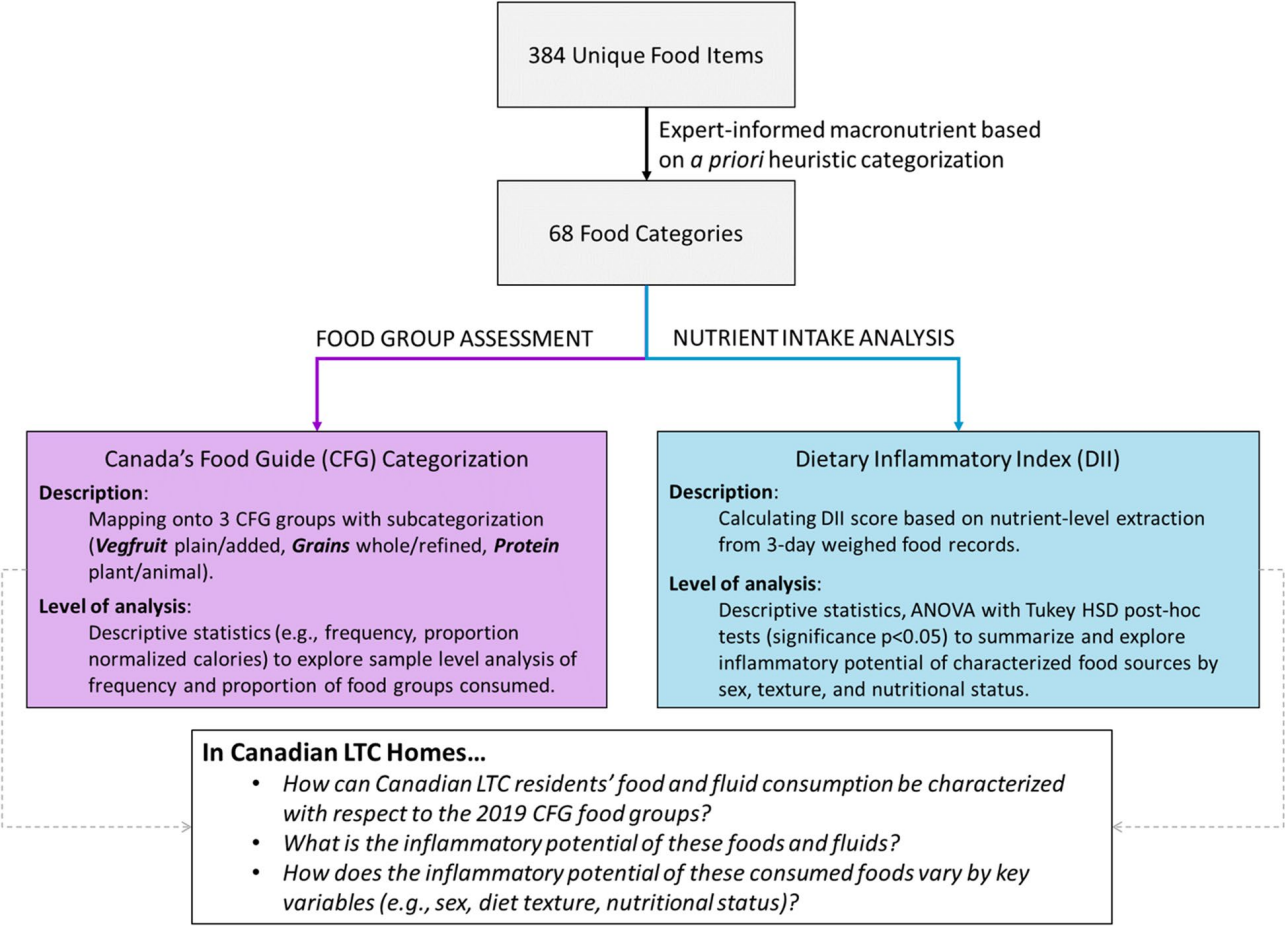
Overview of data flow. Overview of approaches employed to address research questions including the re-categorization of food items in accordance with the 2019 Canada’s Food Guide (CFG) and the Dietary Inflammatory Index

We conducted a secondary analysis of the Making the Most of Mealtimes (M3) dataset that included 3-day weighed food entries of 640 residents living in 32 LTC homes across four provinces in Canada. Details on the data and data collection methods for this dataset have been reported elsewhere [30]. Six residents with implausibly large portions of food items were removed prior to processing resulting in 47 056 food items representing 634 residents’ food and fluid intake records. Figure 1 provides a snapshot of the flow of processed data to address our research questions.

We have categorized food and fluid intake, both described in further detail below. The first based is based on macronutrient content similarity, the second based on standard food group categories from the 2019 CFG. We sought to categorize LTC food source consumption based on expert-informed macronutrient content similarities because there is evidence suggesting menu planning and grouping without considering nutrient content or nutrient density of foods and fluids may be insufficient for addressing needs  [31, 32]. Second, in Canada, provincial regulations govern menu planning in LTC homes [31]. As such, there is merit in also grouping according to the 2019 CFG food groups as this is the approach that has historically informed policy.

### Characterizing food sources with an expert-informed heuristic algorithm

This section describes our process for characterizing food sources for automatic macronutrient-driven groupings. One drawback of characterizing food sources is that food item categorization in large datasets is time consuming and subjective when done by hand [33]. To address this inefficiency, we present a novel algorithm that uses artificial intelligence for auto-categorization based on expert-informed a-priori rules for systematic hierarchical macronutrient-driven food groupings. Given the massive size of the M3 database, we needed an automated categorization approach that enables repeatable, predictable, explainable, and fast processing of food records. We employed a novel expert-informed heuristic algorithm informed by experts to accomplish this task. This expert-informed heuristic algorithm automated food groups categorization based on similar macronutrient composition to characterize consumed food sources in LTC. An expert algorithm applies a defined set of rules to automate a task, in this case, expert-informed food record categorization. Because of their transparency and ability to complete simple tasks in a repeatable fashion, they have been used extensively within healthcare settings for decision supports [34–36].

This set of rules was reviewed by a team of nutrition experts including the M3 team of three academic dietitians, a nurse, and a food scientist. Specifically, food items from 3-day food intake records were categorized into macronutrient-driven groupings. To ensure validity,

1. We reviewed all unique food record items (n=384).
2. We collapsed food categories based on expert-informed similarity of major nutrient content. These collapsed categories were informed and reviewed by the M3 research lead (academic dietitian) (n=68). For example, food items such as “turkey”, “chicken”, “poultry food products” were combined into a “poultry” food category. Similarly, the “bread” category contained items initially labelled as “bun”, “dinner roll”, “bagel”, etc.
3. We used a hierarchical text search strategy to parse through the food record label to automate categorization in a repeatable fashion.
4. We iteratively refined the automated text parsing of the food record label until all unique food items were categorized as intended.

An overview diagram summarizing the categorization flow of the M3 dataset into food categories is found in Table 1 with an exhaustive description of how unique food items were categorized in Supplementary Table S1. From greater than 47,000 food items, 384 unique items were determined, which were categorized into 68 expert-informed food groups that had similar macronutrient content.

### Characterizing food sources using 2019 Canada’s Food Guide

This section describes our process for characterizing food sources based on the 2019 Canada’s Food Guide. After foods were automatically assigned to a food category, we recategorized foods into CFG groupings according to the 2019 CFG using a set of heuristics to better understand how food intake compared to CFG guidelines (i.e., vegetables and fruits (*vegfruit*), *grains*, and *protein source*). CFG 2019 identifies a nutritious diet as having 50% of calories as fruits and vegetables, 25% as whole grains and 25% as lean proteins, including plant-based proteins [8].

To explore potential differences between more and less healthful sources, these categories were further subdivided into: whole vs. refined *grains*; animal vs. plant sources of protein; and healthier preparations vs. *vegfruit* with less healthful ingredient additions (e.g., sugar, breading, salt as described in CFG [8]). For example, unsweetened applesauce was assigned to VEGFRUIT_PLAIN whereas baked apple crisp (a dessert made with a sugar, oatmeal, and butter topping on baked apples) was assigned to VEGFRUIT_ADDED. In terms of protein sources, while similar protein content lead MILK_SOYMILK to be in the same macronutrient-driven food groupings, for the CFG groupings, cow milk sources of protein were re-grouped into animal-based protein while soymilk was re-grouped into plant-based proteins.

While lower-level food categories for the protein source and grains were well defined and easily combined in the algorithm, there was more variability in type of food items and preparation method that fit within the *vegfruit* category. As a result, re-categorization of *vegfruit* items into either the healthier preparation or the added categories were conducted by hand. To be assigned into one of these categories, several of the macronutrient/expert opinion food categories were re-defined. For example, macronutrient-wise, potatoes and grains are similar, however potatoes were categorized as *vegfruit* to align with CFG. Categorizations of each record item also relied on a keyword search through the full intake record to extract preparation methods (e.g., “seasoned” implied salt was added; seasoned carrots were categorized as VEGFRUIT_ADDED). Table 1 examples 1, 2, 3, 6 and 10 refer to protein sources to show the flow into subcategories including how dairy, which previously had its own food group, fits into the new CFG grouping of an animal source of protein; and that legumes are counted as plant sources of protein. Similarly, juice is no longer considered by the 2019 CFG as part of the vegetables or fruits food group, and was thus categorized into the OTHER food group category [8].

**Table 1:** Categorization algorithm examples of 3 day food intake records into Canada’s Food Guide subcategories. This table shows the flow from the 47 054 food entries from 634 resident 3-day weighed food intake records to final categorization into Canada’s Food Guide (CFG) groups

# Day M3 Food Intake Record Examples	Unique Food Item Examples	Canada’s Food Guide Recategorization
1. MILK, FLUID, PARTLY SKIMMED, 2% MILK FAT	1. MILK	1. PROTEIN: animal
2. MAH PO TOFU 38	2. PROTEIN-VEG	2. PROTEIN: plant
3. AB-2-Omelet	3. OMELET	3. PROTEIN: animal
4. BREAD, WHOLE WHEAT, COMMERCIAL, TOASTED	4. BREAD	4. GRAIN: whole
5. STRAWBERRY, HALVES, RAW	5. STRAWBERRY	5. VEGFRUIT: plain
6. TILAPIA-PIER 17	6. TILAPIA	6. PROTEIN: animal
7. DINNER ROLL (WHITE) DLC MB	7. BREAD	7. GRAIN: refined
8. DESSERT, PUDDING, VANILLA, DRY MIX, INSTANT, PREP W/2% MILK	8. PUDDING	8. OTHER
9. POTATO, MASHED, HOMEMADE, 2% M.F. MILK MARGARINE ADD	9. POTATO	9. VEGFRUIT: added
10. PUREE SEASONED GREEN PEAS	10. PEAS	10. PROTEIN: plant
47,054 food entries; 634 residents	384 unique food items	3 food groups; 6 subtypes

### Rationale and implementation of the Dietary Inflammatory Index (DII)

We used the food-group-agnostic Dietary Inflammatory Index (DII) to more directly probe healthfulness of consumed food and fluids. Briefly stated, the DII provides a metric that (1) looks at food and fluid intake, and determines, the nutrients contained in the consumed foods, and (2) based on the nutrient content, calculates an index representing the inflammatory potential of these consumed foods. By emphasizing foods that have lower inflammatory potential, we may be able to ameliorate food’s contribution to inflammation in chronic disease management.

More specifically, Shivappa and colleagues designed the DII based on 6500 articles identifying 45 food parameters (e.g., energy, protein, fibre, n-3 and n-6 fatty acids, cholesterol, vitamin c) associated with 6 pro-inflammatory biomarkers (i.e., IL-I$\beta$, IL-4, IL-6, IL-10, TNF-$\alpha$, and C-reactive protein) [37]. The DII has the advantage of summarizing the whole diet into a single value that indicates potential “healthfulness” of intake, making it attractive for epidemiological studies. A higher score indicates a more pro-inflammatory profile, and negative scores indicate an anti-inflammatory food profile. Prior work has used the DII to describe food intake across sexes, body mass index categories, age groups, physical activity levels  [38]. To date, quality of dietary intake in LTC has been poorly described, although comparison to nutrient requirements has been done [30]; zinc, vitamin K, and vitamin B6 are often poorly consumed in this population and of clinical relevance (e.g., cognition, wound healing, immunity) [30, 39, 40].

Compared to the 45 food parameters outlined by the DII originators [37], we omitted 18 due to data unavailability (eugenol, garlic, MUFA, onion, PUFA, saffron, turmeric, green/blacktea, flavan-3-ol, flavones, flavonols, flavonones, anthocyanidins, isoflavones, pepper, thyme/oregano, rosemary) as well as 4 which were unreliably reported in the M3 dataset (alcohol, caffeine, vitamin D, vitamin E). Specifically, vitamin D and E were incompletely reported within the Canadian Nutrient File, caffeine was not systematically reported, and alcohol consumption was so infrequent that it was removed during data cleaning. As a result, we implemented the DII using 23 parameters available in the M3 nutrient file as outlined in Table 2 with the weights in accordance with Shivappa et al. [37]. The omitted parameters were exclusively anti-inflammatory parameters. Thus, the sum of the pro-inflammatory effect scores did not change from the original DII parameters (1.446 vs. 1.446), but the sum of the anti-inflammatory effect scores was reduced by 60% ($-4.622$ vs. $-11.706$).

#### Calculating a resident’s overall DII score

For each of the 634 residents, their three-day food intake was tallied, and each DII parameter intake was summed and averaged across the three days. For each DII parameter of interest, we calculated the resident’s standardized nutrient intake z-score using the nutrient global daily mean ($\mu _i$) and standard deviation ($\sigma _i$) from a simulated World dietary intake database [37]:

1 $$\begin{aligned} z_{r,i} = \frac{x_{r,i} - \mu _{i}}{\sigma _{i}} \end{aligned}$$

where $x_{r,i}$ is the intake of DII parameter nutrient *i* by resident *r*. This score was then expressed as a percentile ($p_{r,i}$) and subsequently centered around 0 and scaled to be bounded between $-1$ (maximally anti-inflammatory) and 1 (maximally pro-inflammatory):

2 $$\begin{aligned} c_{r,i} = 2p_{r,i}-1 \end{aligned}$$

A resident’s nutrient-specific DII score was then computed by multiplying the centered score by the DII effect score ($\epsilon _i$) [37]:

3 $$\begin{aligned} DII_{r,i} = c_{r,i} \epsilon _i \end{aligned}$$

Finally, the resident’s overall DII score was calculated by summing the food parameter specific scores over all 23 DII food parameters included in this study:

4 $$\begin{aligned} DII_r = \sum _{f_i \in F} DII_{r,i} \end{aligned}$$

where $f_i$ is a food parameter from the set *F* of relevant DII food parameters included in this study (Table 2).

**Table 2:** Summary of average LTC consumption based on the 23 M3 food parameters included in calculating the Dietary Inflammatory Index (DII)

Food Parameter	DII Weight	Canadian RDA/AI**	Mean Resident Intake***
Energy (kcal)	0.18	n/a	1561
Protein (g)	0.021	m 56; f 46	58
Carbohydrate (g)	0.097	130	217
Fibre (g)	-0.663	m 30; f 21	12.7
Total fat (g)	0.298	ND	52.7
Saturated fat (g)	0.373	n/a	15.9
Trans fat (g)	0.229	n/a	1.5
n-3 Fatty acids (g)	-0.436	m 1.6; f 1.1	1.3
n-6 Fatty acids (g)	-0.159	m 14; f 11	7.3
Cholesterol (mg)	0.11	n/a	240
Vitamin A (RAE mcg)	-0.401	m 900; f 700	658
b-Carotene (mcg)	-0.584	n/a	2114
Thiamin/Vitamin B1 (mg)	-0.098	m 1.2; f 1.1	1.3
Riboflavin/Vitamin B2 (mg)	-0.068	m 1.3; f 1.1	1.8
Niacin/Vitamin B3 (mg)	-0.246	m 16; f 14	14.1
Vitamin B6 (mg)	-0.365	m 1.7; f 1.5	1.2
Vitamin B12 (mcg)	0.106	2.4	3.7
Vitamin C (mg)	-0.424	m 90; f 75	104
Folic acid (mcg)	-0.19	400	236
Fe (mg)	0.032	8	9.5
Mg (mg)	-0.484	m 420; f 320	223
Zn (mg)	-0.313	m 11; f 8	7.3
Selenium (mg)	-0.191	55	72

* Overall inflammatory effect score weightings consistent with Shivappa et al. [37]; negative effect scores denote an anti-inflammatory effect whereas the positive effect scores denote a pro-inflammatory effect. ** Canadian Recommended Dietary Allowance (RDA)/Adequate Intake (AI) from Health Canada dietary reference intakes tables for age group >70 years. Where there was a sex difference in RDA/AI requirements, the average was taken; n/a implies they were not listed in the tables.*** Mean resident intake is from food sources only and does not include contributions from any additional micronutrient supplement pills

#### The adjusted DII: rescaling to compare to Shivappa’s 45 parameter DII

We also implemented an “adjusted version” of the DII to facilitate comparison across other populations and studies. The M3 nutrient files for each food item reflects 51% (23/45) of the potential food parameters described by Shivappa and colleagues. For the adjusted DII, we used the sum of the anti- and pro-inflammatory parameters from the original 45 parameters [37], and we rescaled our 23 parameters according to the theoretical total weights with 45 parameters. This adjusted DII score was computed using the grand total pro-inflammatory (1.446) and anti-inflammatory ($-11.706$) effect scores. Specifically, each nutrient was linearly scaled assuming:

5 $$\begin{aligned} \widetilde{DII}_{r} = \sum\limits_{f_i \in F_{anti}} DII_{r,i} \left( \frac{-11.706}{\sum _{f_j \in F_{anti}} \epsilon _j} \right) + \sum\limits_{f_i \in F_{pro}} DII_{r,i} \left( \frac{1.446}{\sum _{f_j \in F_{pro}} \epsilon _j} \right) \end{aligned}$$

where $F_{anti}$ and $F_{pro}$ are the set of anti-inflammatory and pro-inflammatory nutrients in foods. For a given dataset with a set of available food parameters, the two terms in brackets are constants. In this 23-parameter dataset, the scaling factors evaluated to 2.5327 ($-11.706/-4.662$) and 1 (1.446/1.446).

Using the mean M3 resident intake in Table 2 to describe a hypothetical resident, the following provides a worked example of calculating the adjusted DII. The sum of the anti-inflammatory scores before scaling was -1.11, and the sum of pro-inflammatory scores was 2.12. To account for the disparity in the proportion of anti-inflammatory markers as the original 45 set, this hypothetical resident’s anti-inflammatory DII was scaled by 2.5327 to yield an adjusted anti-inflammatory score of -2.81. This hypothetical resident’s total adjusted DII score then becomes -2.81 + 2.12 = -0.69 with a calculated DII of 2.12 - 1.11 = 1.01.

### Diet texture and nutritional status

A total of 47% of the LTC population receives modified texture foods [41]. This is typically prescribed for providing nutrition safely due to swallowing or chewing difficulties [41]. As described in  [41], prescription of modified texture diet was defined based on each individual LTC where each resident’s prescribed texture diet was categorized as either regular, minced meat, minced, puréed or other; nutritional status was defined by the categorical Mini Nutritional Assessment short form (MNA-SF) as either being malnourished, at risk, or normal.

### Statistical analyses

Statistical analyses were conducted in MATLAB R2016b. Descriptive statistics such as frequency and mean percentage of total daily kilocalorie (kcal) were used to summarize the food intake groupings across the sample as well as mean (SD) inflammatory potential across residents and across macronutrient-driven food groupings. Box plots were used to visually summarize differences of inflammatory potential for sex, nutritional status, and diet texture-based comparisons. One-way ANOVA was conducted with Tukey-Kramer post-hoc analysis (Tukey’s honestly significant difference (HSD)) to assess these differences [42]. We considered p<0.05 as statistically significant.

## Results

### Characterization of LTC residents’ food sources

Based on the expert-informed food groupings (68 categories), the top 5 food categories accounting for the highest mean daily intake in LTC (mean daily kcal; % total daily kcal; frequency of consumption/day) were: *juice* (152.6; 9.8%; 2.3 times/day), *bread* (132.2; 8.5%; 1.1 times/day), *milk_soymilk* (121.1; 7.8%; 2.2 times/day), *oral nutritional supplement* (80.1; 5.1%; 0.5 times/day), and *cereal* (74.7; 4.8%; 0.8 times/day). Here the frequency of consumption per day is defined as a theoretical “average” resident (i.e., mean intake across 634 residents). For a full overview and distribution, see Table 3.

**Table 3:** Consumption patterns across all 68 macronutrient-driven LTC food/fluid groupings and the related Canada’s Food Guide food groups ($n_{items}$ = 47 056; $n_{residents}$ = 634)

Expert Informed Group	CFG Group	Daily intake, kcal/day		freq/day
		mean (SD)	%	
JUICE	OTHER	152.6 (106.7)	9.8%	2.3
BREAD	GRAIN, REFINED	132.2 (111.8)	8.5%	1.1
MILK_SOYMILK	PROTEINS, ANIMAL	121.1 (111.7)	7.8%	2.2
ORAL_NUT_SUP	OTHER	80.1 (169.7)	5.1%	0.5
CEREAL	GRAIN, WHOLE	74.7 (60.3)	4.8%	0.8
DESSERT_OTHER	OTHER	71.1 (79.6)	4.6%	0.7
CAKE_LOAF	GRAIN, REFINED	64.3 (81.2)	4.1%	0.3
POTATO	VEGFRUIT, PLAIN	62.7 (50.7)	4%	0.9
SOUP_STEW	PROTEINS, ANIMAL	54.3 (49.8)	3.5%	0.8
EGG	PROTEINS, ANIMAL	52.2 (56.9)	3.3%	0.6
SANDWICH	PROTEINS, ANIMAL	47.8 (75.6)	3.1%	0.3
CANDY	OTHER	46 (46.4)	2.9%	1.2
POULTRY	PROTEINS, ANIMAL	45.7 (51)	2.9%	0.4
BUTTER	OTHER	40.2 (45.3)	2.6%	0.8
BEEF	PROTEINS, ANIMAL	39.6 (50.3)	2.5%	0.3
PORK	PROTEINS, ANIMAL	39.2 (47.9)	2.5%	0.4
PIE	GRAIN, REFINED	32.5 (50.8)	2.1%	0.2
FRUIT_OTHER	VEGFRUIT, PLAIN	30.6 (40.9)	2%	0.6
MUFFIN	GRAIN, REFINED	30.3 (60.9)	1.9%	0.2
COOKIE	OTHER	29 (44.5)	1.9%	0.3
SAUCE	OTHER	24.9 (26.6)	1.6%	0.8
MIXED_DISH_PASTA	PROTEINS, ANIMAL	23 (42.2)	1.5%	0.1
FISH_SEAFOOD	PROTEINS, ANIMAL	20.3 (32.5)	1.3%	0.2
YOGURT_DAIRY_OTHER	PROTEINS, ANIMAL	19 (39.5)	1.2%	0.2
VEG_OTHER	VEGFRUIT, PLAIN	16.3 (17)	1%	0.6
CONDIMENTS_SUGAR	OTHER	15.7 (20.7)	1%	0.5
BANANA	VEGFRUIT, PLAIN	15.2 (26.1)	1%	0.2
CHEESE	PROTEINS, ANIMAL	12.3 (27.1)	0.8%	0.1
MEAT_PROCESSED	PROTEINS, ANIMAL	12.1 (28.8)	0.8%	0.1
BEAN	PROTEINS, PLANT	11.5 (25.4)	0.7%	0.2
PEANUT_BUTTER	PROTEINS, PLANT	10.7 (31.1)	0.7%	0.1
FAST_FOOD_CHIPS	OTHER	10.2 (32.3)	0.7%	0.1
WHOLE_GRAIN	GRAIN, WHOLE	9 (33.5)	0.6%	0.1
PUDDING	OTHER	8.8 (29.3)	0.6%	0.1
CREAM	OTHER	7.8 (23.6)	0.5%	0.4
CASSEROLE_MEAT	PROTEINS, ANIMAL	7.7 (24.6)	0.5%	0
APPLE	VEGFRUIT, PLAIN	7.6 (13.6)	0.5%	0.4
ICECREAM	OTHER	7 (25.4)	0.4%	0.1
CRACKER	GRAIN, REFINED	6.5 (25.5)	0.4%	0.2
PANCAKE	GRAIN, REFINED	6.1 (21.5)	0.4%	0
VEG_CRUC	VEGFRUIT, ADDED	5.7 (11)	0.4%	0.2
FRUIT_CRUMBLE	VEGFRUIT, ADDED	5.1 (20.7)	0.3%	0
CARROT	VEGFRUIT, PLAIN	4.6 (8.8)	0.3%	0.2
CITRUS	VEGFRUIT, ADDED	4.2 (9.9)	0.3%	0.1
THICKENER	OTHER	3.7 (18.4)	0.2%	0.3
BAR	OTHER	3.4 (18.1)	0.2%	0
LEAFY_GREENS	VEGFRUIT, ADDED	3.4 (8.4)	0.2%	0.2
BERRIES	VEGFRUIT, PLAIN	3.3 (8.3)	0.2%	0.1
SODA_DRINK	OTHER	3.2 (10.2)	0.2%	0.1
PEAS	PROTEINS, PLANT	2.8 (7.1)	0.2%	0.1
JELLO	OTHER	2.7 (16.8)	0.2%	0
NOODLES	GRAIN, REFINED	2.6 (18.3)	0.2%	0
FILLED_PASTRY_SAVOURY	OTHER	2.5 (15.9)	0.2%	0
SMOOTHIE	OTHER	2.3 (36.9)	0.1%	0
PUREE_SUPPER	PROTEINS, ANIMAL	1.6 (20.6)	0.1%	0
TEA	OTHER	1.6 (4.8)	0.1%	0.7
WATER	OTHER	1.6 (7.3)	0.1%	1.1
SNACK	OTHER	1.3 (8.8)	0.1%	0
CONDIMENTS	OTHER	1.2 (4.3)	0.1%	0.2
HOT_CHOCOLATE	OTHER	1.1 (18)	0.1%	0
SQUASH	VEGFRUIT, PLAIN	1.1 (3.6)	0.1%	0.1
PROTEIN_VEG	PROTEINS, PLANT	1 (9.7)	0.1%	0
PROTEIN_POWDER	PROTEINS, ANIMAL	0.7 (10.5)	0%	0
TOMATO	VEGFRUIT, ADDED	0.7 (3.9)	0%	0
NUTS_SEEDS	PROTEINS, PLANT	0.6 (4.4)	0%	0
COFFEE	OTHER	0.5 (4)	0%	1.1
ALCOHOL	OTHER	0.4 (3.1)	0%	0
GRANOLA BAR	GRAIN, WHOLE	0.4 (4.8)	0%	0

Mean daily kcal intake represents a theoretical average resident (i.e., mean intake across 634 residents); frequency of consumption is per resident per day for the theoretical average resident (i.e., divided by 634 residents). Coffee and tea were included due to the common practice of additions of milk, cream, and sugar. SD: standard deviation

**Fig. 2:**
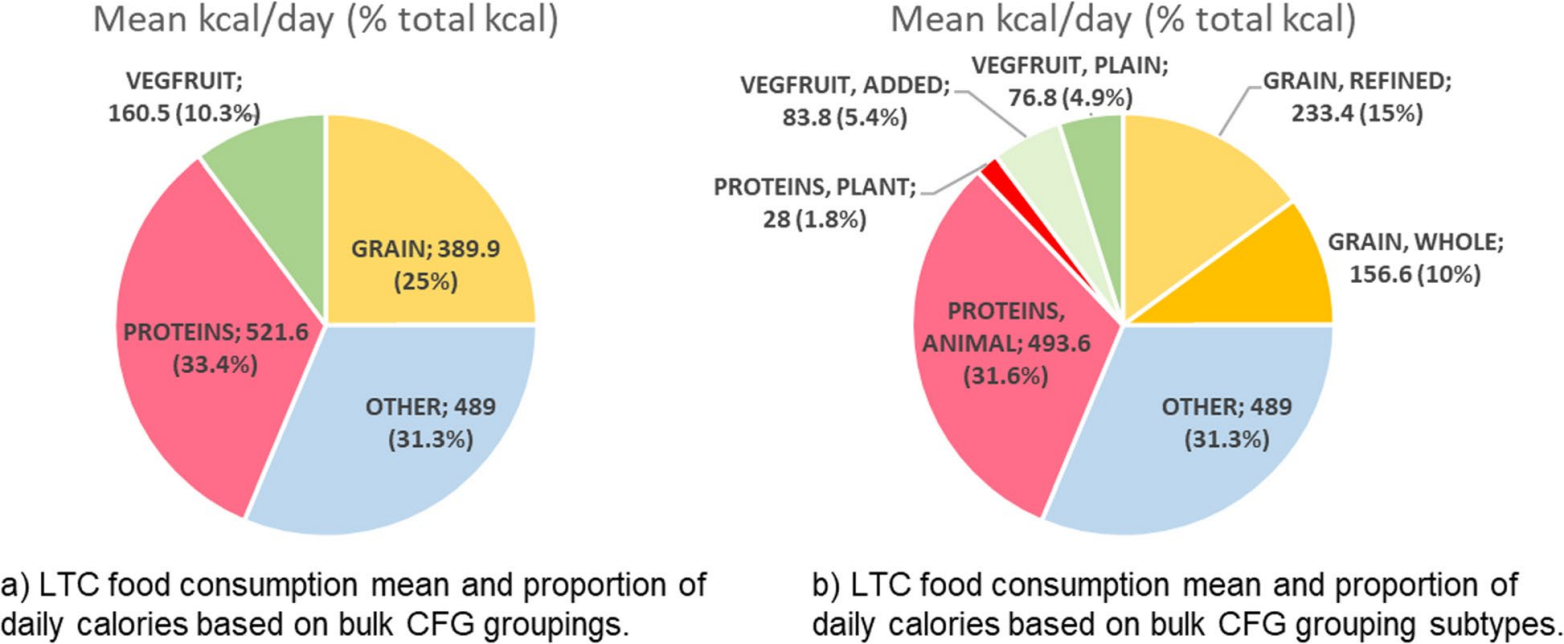
LTC food consumption using Canada’s Food Guide 2019 food groups. LTC food consumption kilocalorie plots based on Canada’s Food Guide three bulk categories of *protein*, *grains*, and vegetables and fruits (*vegfruit*) as well as an OTHER category. **a** Mean kcal consumption and the proportion of kcals (%) of food group intake for each food group; **b** Three bulk food groups are broken down into subtypes reflecting more and less healthful sources (e.g., plain vegfruit vs. vegfruit with added components like sugar, salt, or extra breading as per Canada’s Food Guide [8]). These proportions by frequency and calories are based on 47 056 food intake records from 634 residents

**Fig. 3:**
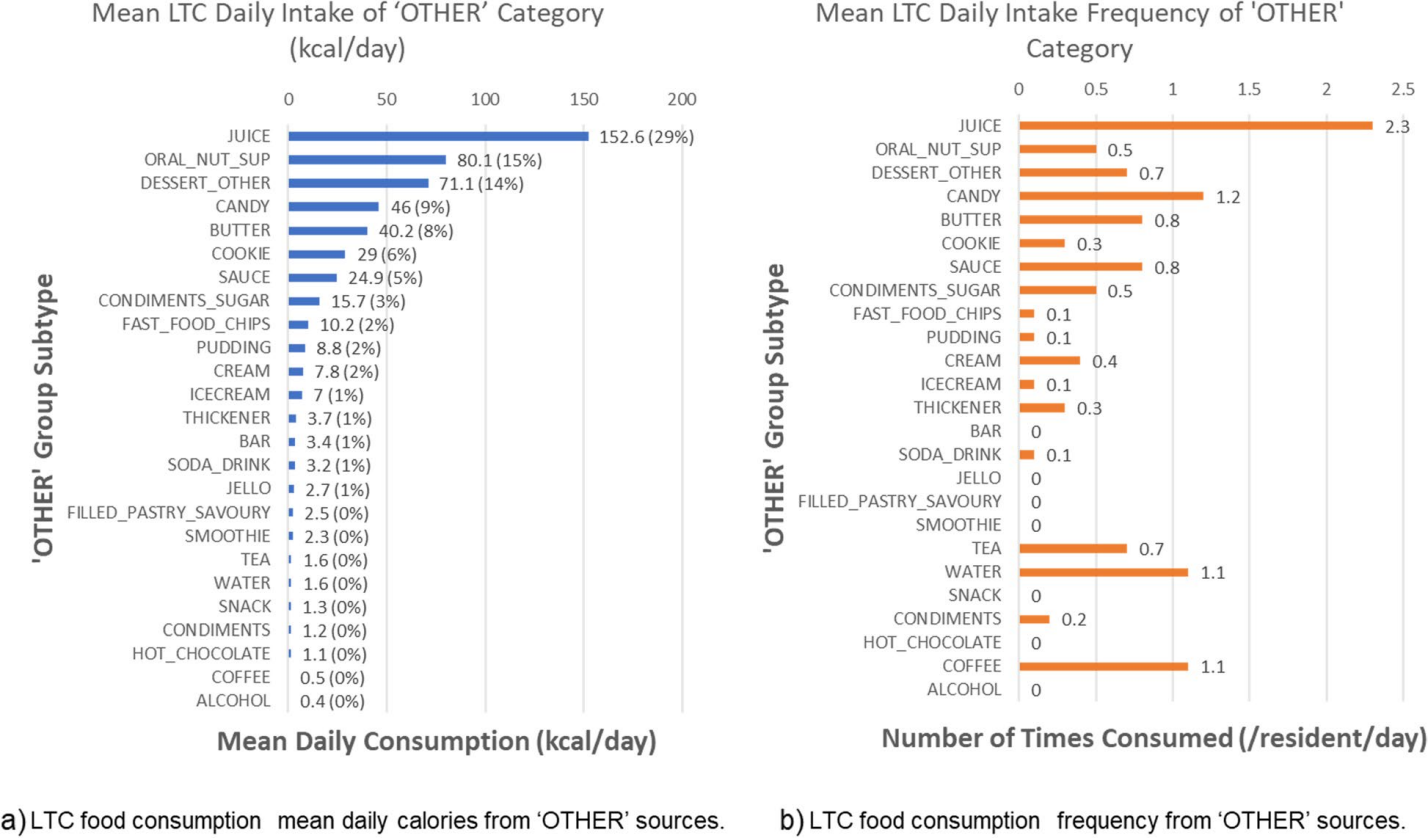
OTHER foods composition. LTC food consumption OTHER category distribution shown as a) calorie and b) frequency plots for food consumption in LTC. These frequencies and proportion calories are based on 47 056 food intake records from 634 residents. This OTHER category was defined through omission of the item in Canada’s Food Guide (i.e., all categories which did not fit under *vegfruit*, *protein*, or *grains* [8])

Based on the CFG re-categorization of these food groupings (Fig. 2), we presented intake of (*grains*, *vegfruit*, *protein sources*) both by mean calories (kcal) consumed as well as the proportion of total calories consumed across the M3 LTC sample. A much smaller proportion of consumed calories came from *vegfruit* (10.3%; 4.9% plain) and *grains* (25.0%; 15.0% refined grains) compared to *protein sources* (33.4%; 31.6% animal source) and OTHER food groups (31.3%). The substantial contribution from the OTHER category (31.3% by kcal) reflects that many frequently consumed foods do not fit neatly into the CFG 2019 food group categories. Specifically, the top-5 categories contributing to the OTHER category were: juice (29% of OTHER, 9.8% by kcal), oral nutritional supplements (15% of OTHER, 5.1% by kcal), dessert-other (14% of OTHER, 4.6% by kcal), candy (9% of other, 2.9% by kcal), and butter (8% of OTHER, 2.6% by kcal). For further assessment of contributing groups to the OTHER category, see Fig. 3.

**Fig. 4:**
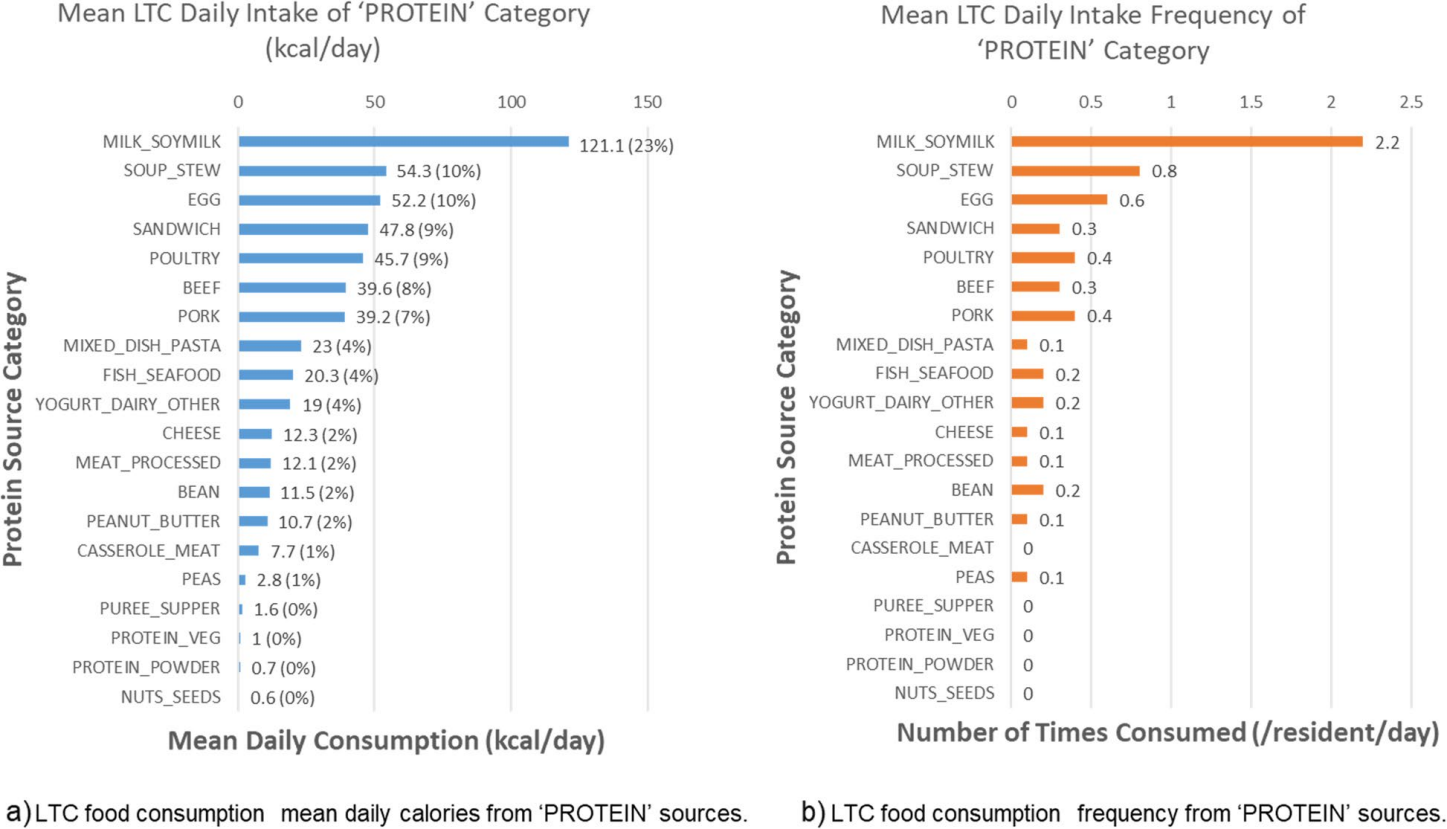
Protein intake sources. Protein intake sources shown as **a** mean daily kcals of protein consumed by each of the protein categories (**blue**) and **b** relative frequency of consumption of that protein intake source based on 47 056 food items and 634 residents’ intake (orange)

As protein intake is seen as one of the most important nutrients of concern in older adults to prevent frailty [43], we conducted a further assessment of consumed protein sources based on mean consumption across the sample ($n_{items}=47~056$; $n_{residents}=634$) as shown in Fig. 4. The majority of protein intake by calories was through milk/beverages (dairy or soy) (23%), followed by soups and stews (10%), eggs (10%), sandwiches (9%) and poultry sources (9%). Regarding proportion of protein sources in the context of all CFG food groups, Fig. 2 highlights 31.6% of mean consumed calories were from animal-based proteins while only 1.8% were consumed from plant-based proteins. Supplementary Table S2 provides additional details on the average energy (kcal) and protein intake of the sample based on direct nutrient consumption from food sources by subgroup.

### LTC foods sources and their inflammatory potential

The DII scores provide a food healthfulness perspective complementary to the evidence-based CFG. Recall that for the 23 food parameters included, the total inflammatory and anti-inflammatory weights summed to 1.446 and $-4.622$ (60% reduction from the original $-11.706$ overall anti-inflammatory score), respectively. This implies for this dataset there is an implicit bias towards inflammatory (positive) DII scores compared to the initial 45-parameter scores [37]. Table 4 shows the maximum and minimum adjusted DII scores were bounded between $-7.01$ and +8.36 with a median of 2.76 and positive value at the 25th percentile (0.57). Therefore, M3 data food intake is skewed towards positive, pro-inflammatory values.

**Table 4:** Summary of inflammatory potential of Canadian LTC food consumption, an adjusted version to account for a different number of food parameters included, and the simulated world DII described by Shivappa et al. [37]

Summary Statistic	M3 data DII	Adjusted M3 data DII	Simulated World DII (Shivappa et al 2013)
Maximum	3.30	8.36	7.98
90th percentile	2.25	5.71	4.00
75th percentile	1.75	4.43	1.90
Mean	0.93	2.35	not reported
Overall median	1.09	2.76	0.23
25th percentile	0.23	0.57	$-2.36$
10th percentile	$-0.74$	$-1.88$	$-3.37$
Minimum	$-2.77$	$-7.01$	$-8.87$
# food parameters	23	23 (45 equivalent)	45
# participants	634	634	n/a

#### Effect of covariates on consumed foods’ inflammatory potential

**Fig. 5:**
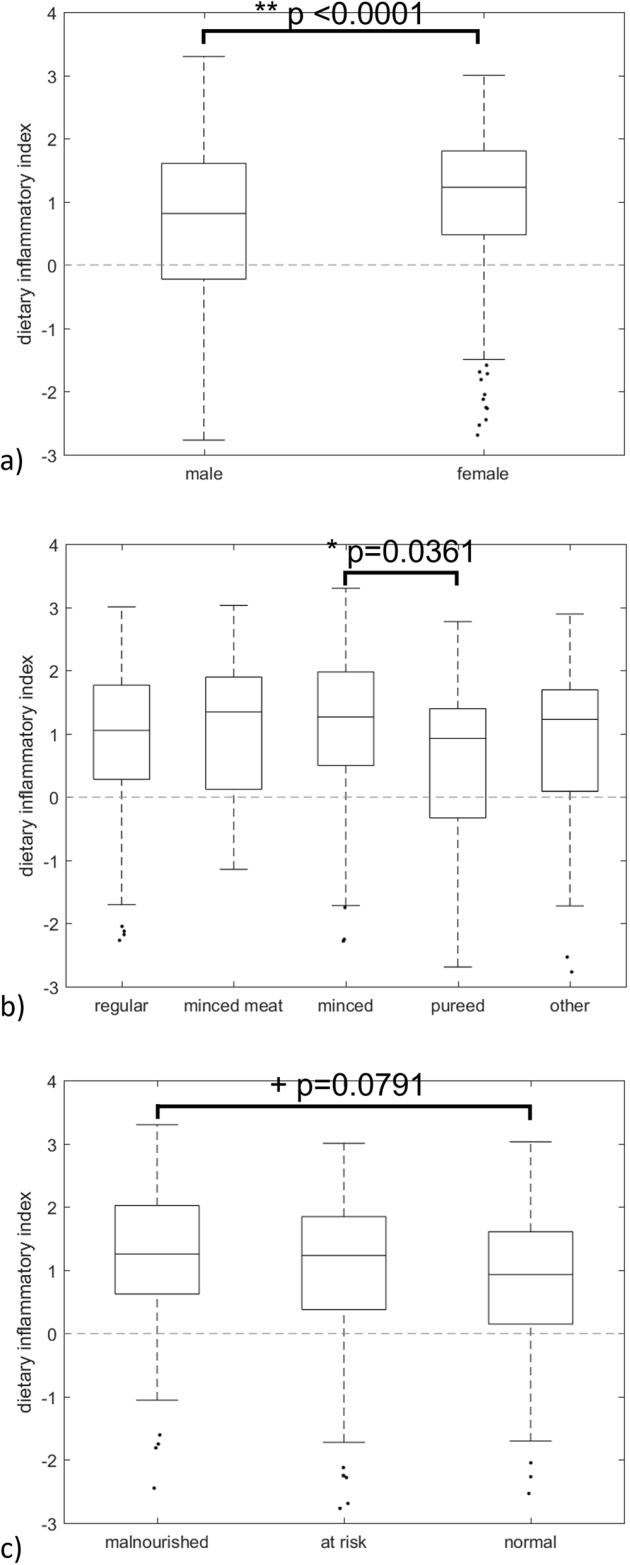
How DII scores differ by key variables. DII scores and their relationship to **a** sex, **b** texture, and **c** nutritional status

As shown in Fig. 5 there were significant differences (p<0.0001) in DII scores for sex and diet texture (see Table 5 for supporting statistics and Supplementary Table S3 for full ANOVA output).

**Table 5:** Covariate dietary inflammatory index and sample demographics

Variable	Group	Mean DII	SD	n	%
*Sex (n=634)*	Male	0.66	1.25	195	30.8%
	Female	1.05	1.07	439	69.2%
*Texture (n=634)*	Regular	0.97	1.08	327	51.6%
	Minced meat	1.09	1.24	20	3.2%
	Minced	1.14	1.19	84	13.2%
	Pureed	0.57	1.28	63	9.9%
	Other	0.86	1.15	140	22.1%
*Nutritional Status (n=633)*	Malnourished	1.16	1.22	80	12.6%
	At Risk	0.97	1.18	261	41.2%
	Normal	0.84	1.06	292	46.1%

Regarding sex differences, A one-way ANOVA was performed to compare the effect of sex on DII score which revealed a statistically significant difference in DII score (F(1,632) = 16.4, p<0.0001). Tukey’s HSD test for multiple comparisons found that the mean DII value was significantly different between males and females (p<0.0001, 95% CI = [-0.585, -0.203]).

Regarding texture, a one-way ANOVA was performed to compare the effect of diet texture on DII which revealed a statistically significant difference in DII (F(4,629) = 2.59, p=0.0361). More specifically, Tukey’s HSD test for multiple comparisons found that the mean DII value was significantly different between minced and puréed texture diets (p=0.02, 95% CI= [0.051, 1.086]) but no significant difference between other diet texture subgroups (p>0.8).

Nutritional status as defined by the categorical MNA-SF assessment showed that malnourished residents consumption had higher DII scores (higher inflammatory potential) than those of normal nutritional status. However, based on the one-way ANOVA, there were no statistically significant differences between groups (p=0.079).

## Discussion

### Principle findings

This work describes, a tool and infrastructure to assess resident intake at the home-level may provide a strategy for data-driven insights for menu planning to support nutritional value of food. Our three main take-aways were as follows.

The first key take-away is that protein sources of food in LTC substantially rely on animal-sources which increase inflammatory potential of the diet. The most common source of animal based protein was fluid milk, followed by soups and stews. While this is in opposition to the 2019 CFG recommendation to encourage more plant-based sources of protein [8], it is consistent with LTC emphasizing foods with high acceptability as well as nutrient density (i.e., higher protein content for smaller portion size). In terms of proportion of daily calories coming from protein, Dietitians of Canada recommend for LTC residents to have an acceptable macronutrient distribution range AMDR of 10-35% of daily calories, or an average of 100 g provided on the daily menu [44]. Although this may meet the RDA for several individuals, it does not meet recommendations focused on preventing sarcopenia at 1.2- 1.6 g/kg body weight per day [45]. Oral nutritional supplements, categorized as ’other’ for the comparison to CFG, was relatively high and necessary to meet the potential short-fall in protein intake. Balancing the healthfulness of the diet, as determined by the DII or CFG, with protein adequacy demonstrates the complexity of menu planning in LTC. More boadly, as evidenced by feasibility of nutrient enhanced foods in residential care, additional work to better understand acceptability and palatability of alternative, more sustainable, plant-based protein sources may be beneficial [46].

The second key take-away in this portion of the analysis is that the vegetables and fruit category consumption was much less than the 50% recommended by CFG, indicating an opportunity to improve menu planning and nutrient density as well as decreasing the pro-inflammatory nature of the diet. Juice was the most frequently consumed single item with average consumption of more than 2 times per day. Yet, the new CFG removed juice from this category as it was considered a less healthful option, being high in natural and potentially added sugars. Due to the high consumption of juice in LTC (see Fig. 3) this shifted the calorie consumption to the OTHER food grouping in the 2019 CFG.

The third key take-away from the CFG comparison is that total grain consumption in LTC is aligned with the 2019 CFG guidelines, 25% of calories consumed. However the type of grain matters; more refined grains were consumed by residents in this sample (10% whole; 15% refined) resulting in reduced nutrient density as well as increased pro-inflammatory potential. The practical implication of this consumption pattern reinforces a lower fibre intake that is below the RDA recommendation [30]. As with fruit and vegetable intake, resident preferences and home budgetary and food purchasing constraints influence these offerings.

### Practical implications

This work suggests that the healthfulness of the LTC diets need to be improved. Specifically, refined grains, less healthful vegetables and fruits (i.e., consumed with added salt or sugar), and a high degree of animal-based proteins were consumed by this sample. These choices are in opposition to the 2019 CFG recommendation of consuming more nutrient dense (i.e., more nutrients for calories in product) and healthful options. Consequently, the DII was pro-inflammatory. There are several drivers of menu planning in LTC that are challenging to circumvent to provide a more healthful diet. Resident preference, quality of life, and the endemic nature of poor food and fluid intake in LTC necessitate a more balanced approach of providing nutrient-dense and energy-dense foods and fluids that residents with eating challenges, dysphagia and poor appetite will eat. Further work on this is needed to understand the nuances of the challenges with menu planning in the LTC context to support more “healthful” options. Data-driven metrics like the DII may provide support for optimising these parameters during menu planning. General observations through this work further illuminated the following opportunities to enhance quality (via reduced DII) during menu-planning.

- Juice is well-received by residents and is an important contributor to “energy and nutrient intake for residents” but should be considered as contributing to fluid intake as opposed to solely a food group [44]
- Grain sources can continue to shift from refined to whole to reduce their contribution to the DII.
- Plant-based protein sources could reduce DII and extend the protein in mixed dishes, stews and soups or baked products [46] but needs to consider product acceptability and comparative protein content of replacements to mitigate concerns for protein malnutrition and sarcopenia [47, 48].
- Desserts are well positioned as potential for enhancement to increase nutritional density (e.g., changing fruit based pie to a fruit cobbler or crumble).

Preferences to promote quality of life are an important consideration in menu planning [49]; LTC is a special population where the focus of care is on quality of life and not the extension of life [44]. The bottom line is that there are multiple layers of complexities when optimising foods aligned with the CFG, shifting to foods with anti-inflammatory potential for enhanced health benefits, while balancing food and fluid offerings which supports a sufficient energy and protein to prevent malnutrition. Appropriately designed education for residents around healthy food choices may also help facilitate synergistic behaviour change [50].

## Limitations

Regarding limitations around available parameters for DII calculation, due to data unavailability, several anti-inflammatory markers were omitted from the DII calculation. While this skewed intake towards pro-inflammatory DII scores, the CFG intake distribution analysis also indicates that the diet consumed by LTC residents requires improvement to be more nutrient dense and healthy. To address this limitation, we presented an adjusted DII score to support comparison to other populations using varying numbers of parameters for the purpose of hypothesis generation. Using the adjusted DII, we saw the mean DII increased compared to the traditional DII. This emphasizes that residents who consumed less anti-inflammatory nutrients compared to the global average defined by Shivappa et al. [37], yielded an amplified pro-inflammatory contribution. This is consistent with the pro-inflammatory mean DII in our data. Likewise, residents with the highest 23-parameter DII scores were amplified even higher from lack of anti-inflammatory nutrient intake. Similarly, those who consumed high anti-inflammatory nutrients attained an even lower adjusted DII score.

The adjusted DII score provides a scaling factor that preserves the original distribution (with fewer parameters), over a wider range of values and centered around percentiles of global intake described by Shivappa et al. [37]. In the M3 data, while the total number of food parameters was lower than described by the originators [37], the M3 dataset contained all of the original pro-inflammatory markers. For context Hébert and colleagues state the bounds typically range from $-8.87$ to +7.98 and rarely exceed 11, or between $-5.5$ to +5.5 on a lower number of parameters (i.e., 25-30) [38]. Our adjusted DII was contained within those typical bounds falling between $-7.01$ and 8.36 with a of median 2.76. The adjusted DII findings should be interpreted with caution as this approach differs from the intended use and may not reflect the true inflammatory potential. Further work validating the adjusted DII score on the same dataset including varied parameters is warranted.

Regarding protein adequacy, while we characterized food sources of protein, based on our assessment of food sources by CFG groups, oral nutritional supplements were considered as part of the other category, and not directly contributing to protein intake. While an in-depth assessment of protein adequacy in this sample was outside the scope of this paper, it is of high relevance in LTC populations with such a high prevalence of sarcopenia and frailty [51]. As such, specific contributions of protein intake by food source should be considered as part of future work. Finally, the expert-informed macronutrient categorization used here to describe frequency of foods consumed, may have resulted in some error in classification, as well as the rules and algorithm to attribute foods to CFG groupings. Yet, the three different methods of summarizing food intake in this sample resulted in consistent conclusions on healthfulness of the diet.

## Conclusion

This paper characterizes Canadian LTC food sources and outlines their dietary inflammatory potential. It provides a new way of automating laborious coding methods for repeatable assignment of food records into categories based on expert-informed categories, and in accordance with Canada’s Food Guide food groups. This paper also characterizes the inflammatory potential of the consumed diet. The foods consumed by this sample were found to be pro-inflammatory with significant differences between sexes (females consumed higher inflammatory potential foods) and diet texture (residents consuming puréed foods had lower inflammatory potential compared to minced foods). Future directions should include more formal analysis on dietary patterns with particular emphasis on exploring sex-based differences. Additionally, comparison of the DII with other established scores to further elucidate opportunities for data-driven insights to support menu planning (e.g., Healthy Eating Index, Mediterranean scores, DASH diet, and Nutrient Rich Foods scores).

## Supplementary Information

**Additional file 1.**

## Data Availability

The de-identified data analysed during the current study are available upon reasonable request. For more information on the process for approval to access this data, contact Professor Heather Keller (hkeller@uwaterloo.ca).

## Abbreviations

CFG: Canada’s Food Guide
DII: Dietary Inflammatory index
LTC: long-term care
M3: Making the most of mealtimes study
MNA-SF: mini nutritional assessment - short form
