# Supplementary material for: Characterizing Canadian long-term care home consumed foods and their inflammatory potential: a secondary analysis
Source: BMC Public Health. 2023 Feb 6;23:261. doi: 10.1186/s12889-022-14934-8 (PMC9903425; doi:10.1186/s12889-022-14934-8)
Supplement: Supplementary file 1 — Additional file 1. [file 12889_2022_14934_MOESM1_ESM.pdf]

Additional Files and Supplementary Information

S1. Macronutrient Driven Food Grouping Summary
This supplementary file provides an overview of how each unique food item was combined based on expert-informed macronutrient-driven groupings.

S2. Resident sample demographics based on daily energy and protein by subgroup
This supplementary file provides further details about sample demographic.

S3. ANOVA results analysing DII scores by sex, texture, and nutritional status
This supplementary file provides further details about the ANOVA statistics for key covariates.

Table S1: Overview of starter grouping and how each food item category was coded into macronutrient-driven food categories.

Group	Contains	Comments
**STARTER GROUPINGS**	’CABBAGE LEAVES_STUFFED’, ’SOUP’, ’PUDDING’, ’CHICKEN’, ’FISH’, ’STEW’, ’CAULIFLOWER’, ’RICE’, ’JUICE’, ’FRUIT’, ’GRAVY’, ’BEAN’, ’OMELET’, ’OMELETTE’, ’SHAKE’, ’CAKE’, ’ICE CREAM’, ’VEGETABLES’, ’JAM’, ’MACARONI’, ’SANDWHICH’, ’SANDWICH’, ’BRUSSEL SPROUT’, ’BRUSSELS SPROUT’, ’CABBAGE ROLL’, ’MUFFIN’, ’COOKIE’, ’BROWNIE’, ’COLESLAW’, ’CHOWDER’, ’PORK’, ’CREAM OF WHEAT’, ’CR OF WHEA’, ’CUCUMBER’, ’MASHED POTATO’, ’DOUGHNUT’, ’EGG’, ’MASH POTATO’, ’FRENCH FRIES’, ’FRENCH TOAST’, ’GRAPE’, ’GRILLED CHEESE’, ’HAM’, ’BEET’, ’HOT DOG’, ’COFFEE’, ’WATER’, ’STEAK’, ’BEEF’, ’CARROT’, ’LASAGNA’, ’KIELBASA’, ’SQUARE’, ’LETTUCE’, ’MANDARIN’, ’TOMATO’, ’CASSEROLE’, ’SCONE’, ’SALMON’, ’SPAGHETTI’, ’COBBLER’, ’BLUEBERR’, ’PEROGIES’, ’PEROGY’, ’VEAL’, ’LIVER’, ’BROCCOLI’, ’CORN’, ’NOODLES’, ’CHILI’, ’CHILLI’, ’MEATLOAF’, ’LOAF’, ’VEGETABLE’, ’MELON’, ’PEACH’, ’PEAR’, ’FETTUCINI’, ’MEATBALL’, ’TURKEY’, ’SHEPHERD"S PIE’, ’SHEPHERD’’S PIE’, ’SHEPHERDS PIE’, ’OATBRAN’, ’OATMEAL’, ’CRISP’, ’PEAR’, ’POTATO’, ’PIZZA’, ’BANANA BREAD’, ’BREAD’, ’SAUSAGE’, ’QUICHE’, ’SPINACH’, ’GELATIN’, ’TOSSED SALAD’, ’SQUASH’, ’YOGURT’, ’ZUCCHINI’, ’GARDEN SALAD’, ’GREEK SALAD’, ’CAESAR SALAD’, ’GREEN SALAD’, ’TOSS SALAD’, ’TOSSED GARDEN SALAD’, ’ASPARAGUS’, ’BACON’, ’BAKED APPLE’, ’BORSCHT’, ’TART’, ’COTTAGE CHEESE’, ’COUSCOUS’, ’FROZEN DESSERT’, ’CHEESE SAUCE’, ’ENSURE’, ’MILK’, ’OAT BRAN’, ’JELLY ROLL’, ’BUN’, ’MANGO’, ’PARSNIP’, ’TURNIP’, ’CRUMBLE’, ’RHUBARB’, ’BAR’, ’SOLE’, ’STRAWBERR’, ’CABBAGE’, ’TUNA SALAD’, ’TOURTIERE’	Looked for these words in the food item description. They were classified in order of where these strings were found. For example, beef barley soup would be classified as soup because ’SOUP’ comes before ’BEEF’.
**ALCOHOL**	’WINE’, ’ALCOHOLIC’	
**SMOOTHIE**	’SMOOTHIE DRINK’, ’SMOOTHIE	
**ORAL NUTRITIONAL SUPPLEMENT (ORAL_NUT_SUP)**	’BENEPROTEIN INSTANT PROTEIN POWDER’, ’BOOST CANADA’, ’RESOURCE 2.0’, ’RESOURCE 2.0 CANADA’, ’ENSURE’, ’PROSOURCE NO CARB LIQUID PROTEIN NS’, ’DRINK_BOOST PLUS’, ’DRINK_BOOST’, ’DRINK_INSTANT BREAKFAST’, ’DRINK_RESOURCE 2.0’, ’FORMULA_ENSURE PLUS’, ’FORUMLA_GLUCERNA’, ’FORMULA_TWOCAL HN’, ’FORMULA’, ’SYSCO MED PLUS 2.0 CANADA’, ’SHAKE’, ’BLENDED BREAKFAST’, ’FORMULA_GLUCERNA’, ’DRINK_BOOST GLUCOSE CONTROL’	

**SODA_DRINK**	'CARBONATED DRINKS', 'DRINK', 'LEMONADE', 'LEMONADE WITH ASPARTAME', 'SODA', 'DRINK_ORANGE FLAVOUR', 'DRINK_LEMONADE FLAVOUR', 'DRINK_CLASSIC ORANGE', 'DRINK_BREEZE', 'DRINK_ORANGE'	
**JUICE**	'CRANBERRY HONEY THICK', 'HT LEMONADE', 'JUICE', 'ORANGE HONEY THICK'	JUICE was from the starter group. So apple juice and orange juice would be captured under juice.
**HOT_CHOCOLATE**	'HOT CHOCOLATE'	
**CREAM**	'CREAM', 'CREAM SUBSTITUTE'	
**APPLE**	'APPLE', 'APPLESAUCE', 'APPLE SAUCE', 'APPLES AND CINNAMON', 'AMBROSIA SALAD', 'BAKED APPLE', 'FORTIFIED APPLESAUCE', 'POMEGRANATE APPLESAUCE', 'CINNAMON APPLES', 'APPLE BROWN BETTY', 'SCALLOPED APPLES'	
**BANANA**	'BANANA', 'PUREE BANANA', 'PUREE BANANA', 'BUREED BANANA', 'PUREED BANANA', 'PUREED BANANA'	
**BEAN**	'BEAN', 'FALAFEL', 'MEATLESS'	BEAN contains: winged beans, weiners and beans, whole green/yellow beans, yellow beans
**BEEF**	'VEAL', 'LIVER', 'BEEF', 'STEAK', 'MEATBALL', 'MEATLOAF', 'TRI PATTIE', 'TRI PATTIE'	
**BERRIES**	'STRAWBERR', 'BLUEBERR', 'CRANBERRY', 'RASPBERRY', 'BLACKBERRY'	
**BREAD**	'BREAD', 'BUN', 'DINNER ROLL (WHITE)', 'ROLL', 'ROLL WW', 'CHEESE BISCUIT', 'CROISSANT', 'GARLIC TOAST', 'BAGEL', 'DRESSING', 'PUREED WHEAT TOAST', 'READ', 'SCONE', 'WHOLE WHEAT ROLL', 'SAVOURY APPLE DRESSING (RE)', 'STRATA LUNCHEON STUFFING'	
**BUTTER**	'MARGARINE', 'BUTTER'	
**CAKE_LOAF**	'BANANA BREAD', 'BROWNIE', 'CAKE', 'LOAF'	

**CANDY**	’CANDIES’, ’CANDY’, ’SWEETS’, ’CHOCOLATE’
**CASSEROLE_MEAT**	’SHEPHERD”S PIE’, ’SHEPERD”S PIE (RE)’, ’SHEPHERD”S PIE’, ’SHEPHERDESS PIE’, ’SHEPHERDS PIE’, ’SHERPHERD”S PIE’, ’SHEPHERDESS PIE’, ’ SHERPHERD”S PIE’, ’CABBAGE ROLL’, ’HILDA”S MEAT PIE’, ’LAMB BOURGUIGNON’, ’MOUSSAKA’, ’ SHERPHERD”S PIE’, ’SHEPHERDESS PIE’, ’TOURTIERE’, ’CHEESEBURGER PIE’, ’PUREED STUFFED BELL PEPPER’, ’CABBAGE LEAVES_STUFFED’
**MIXED_DISH_PASTA**	’BRAISED NAPPA WITH CELLOPHANE NOODLE AND DRIED SHRIMP’, ’CANNELLONI’, ’CASSEROLE’, ’CHEESE CAPPELLETI’, ’CHEESE RAVIOLI’, ’CHEESE TORTELLINI WITH ROSE SAUCE’, ’CREAMY PASTA SALAD SN MB’, ’FETTUCCINE ALFREDO (RE)’, ’FETTUCCINE PRIMAVERA’, ’MACARONI’, ’PASTA PRIMAVERA’, ’PASTA’, ’PASTA SALAD’, ’PASTA SALAD’, ’PUREED BRAISED NAPPA WITH CELLOPHANE NOODLE AND DRIED SHRIMP’, ’ROTINI CHEESE BAKE’, ’SHRIMP PASTA SALAD’, ’TUNA PENNE BAKE’, ’TUNA PESTO SALAD’, ’LASAGNA’, ’SPAGHETTI’, ’PIZZA’
**CEREAL**	’CR OF WHEA’, ’CREAM OF WHEAT’, ’COLD BREAKFAST PUREE’, ’HIGH CALORIE CEREAL’, ’OATBRAN’, ’OAT BRAN’, ’OATMEAL’, ’SUPERCEREAL’, ’WEETS’, ’SEAFOOD CONGEE’, ’DRIED BOK CHOY & DRIED OYSTER CONGEE’, ’BIRD NEST WITH MINCED CHICKEN CONGEE’, ’CONPOY WITH MINCED CHICKEN CONGEE’, ’DRIED BOK CHOY & DRIED OYSTER CONGEE’, ’DRIED FISH & PEANUT CONGEE’, ’MINCED CHICKEN CONGEE W/ DRIED LONGAN’, ’MINCED FISH CONGEE’, ’MINCED FISH CONGEE WITH CILANTRO’, ’MINCED PORK AND PRESERVED EGG CONGEE’, ’MINCED PORK CONGEE WITH LYCII BERRY’, ’PUREED MINCED FISH CONGEE WITH CILANTRO’, ’SEAFOOD CONGEE’, ’TARO MINCED PORK CONGEE’
**CHEESE**	’CHEESE’, ’CHEESE PRODUCT’
**CITRUS**	’CLEMENTINE’, ’LEMON’, ’MANDARIN’, ’ORANGES’, ’TANGERINE’, ’ORANGES’, ’ORANGE’, ’ORANGE SECTIONS’
**CONDIMENTS**	’HORSERADISH’, ’KETCHUP’, ’PICKLE RELISH’, ’PICKLES’, ’SAUERKRAUT’, ’SOY SAUCE’, ’SEASONING’, ’SALT’, ’SPICES’, ’SPREAD’, ’HUMMUS ROASTED RED PEPPER’
**CONDIMENTS_SUGAR**	’ALMOND JELLY’, ’CHOCOLATE SYRUP’, ’CRANBERRY SAUCE’, ’DESSERT TOPPING’, ’JAM’, ’JELLY’, ’MARMALADE’, ’RAISIN SAUCE’, ’SUGAR’, ’SWEETENER’, ’TOPPING’
**COOKIE**	’COOKIE’, ’BISCUIT’, ’RAISIN HERMIT’

**CRACKER**	'CRACKER', 'RACKER', 'MATZOH MEAL'	
**DESSERT_OTHER**	'CHERRY WHIP', ''CREAM PUFF', 'DESSERT', 'DESSERT TOPPING', 'DOUGHNUT', 'ECLAIR', 'ENGLISH TRIFUL', 'ICING (FROSTING)', 'JELLY ROLL', 'JELLY WHIP', 'LEMON BLOSSOMS', 'LEMON CURD', 'PASTRY', 'PINEAPPLE VANILLA PARFAIT', 'PUREE APPLE CINNAMON CUSTARD', 'PUREED LEMON BLOSSOM', 'SQUARE', 'SWEET ROLL', 'TRIFLE', 'TRIFLE', 'ENGLISH TRIFLE'	
**EGG**	'OMELET', 'OMELETTE', 'FRITTATA', 'PUREED OMELET', 'QUICHE', 'PUREED OMELET', 'EGG', 'PUREED OMLETE	
**FAST_FOOD_CHIPS'**	'FRENCH FRIES', ' POUTINE RAPÉE', 'CHIPS'	
**FILLED_PASTRY_SAVOURY**	'PEROGY', 'PEROGIES', 'DUMPLING', 'SAMOSA', 'SPRING ROLL', 'DUMPLING'	
**FISH_SEAFOOD**	'COD', 'TUNA', 'FISH', 'HADDOCK', 'PAN FRIED HADDOCK SITE 22', 'PERCH', 'POLLOCK', 'PUREED COD NUGGETS', 'BAKED POLLOCK', 'FRIED HADDOCK', 'HERRING', 'MEDITERRANEAN POLLOCK FILLET', 'OVEN BAKED COD', 'PAN FRIED HADDOCK', 'SALMON', 'SOLE', 'TILAPIA', 'TROUT', 'STIR FRIED SCALLOPS WITH LILY FLOWER', 'SHRIMP', 'SEAFOOD PLATE', 'PUREED STIR FRIED SCALLOPS WITH LILY FLOWER', 'PUREED SHRIMP', ' MORUE EN SAUCE', 'MOLLUSKS', 'LOBSTER', 'CRAB', 'TUNA SALAD', 'TILAPIA_PIER 17	
**FRUIT_CRUMBLE**	'COBBLER', 'CRISP', 'CRUMBLE', 'RHUBARB'	Rhubarb was included here because it was always cooked with sugar.
**FRUIT_OTHER**	'CHERRY', 'CHERRY WHIP', 'CHILLED APRICOTS', 'FRUIT', 'GRAPE', 'MELON', 'MANGO', 'PAPAYA', 'PEACH', 'PEAR', 'PINEAPPLE', 'PRUNE', 'PRUNE PUREE', 'PRUNES', 'PUREE APRICOT', 'PUREE APRICOTS', 'PUREE PINEAPPLE', 'PUREED CANTELOUPE', 'RED DATES', 'WALDOLF CREAMY APPLE SALAD', 'WALDORF SALAD', 'APRICOT', 'AVOCADO', 'BABYFOOD'	"BABYFOOD" was always a fruit variety.
**ICECREAM**	'ICE CREAM', 'SHERBET', 'FROZEN DESSERT', 'SORBET'	
**JELLO**	'GELATIN', 'LEMON JELLIED SALAD', 'MELLO JELLO', 'WHIPPED JELL-O *'	
**JUICE**	'CRANBERRY HONEY THICK', 'HT LEMONADE', 'JUICE', 'ORANGE HONEY THICK'	

**LEAFY_GREENS**	'GARDEN SALAD', 'GREEK SALAD', 'CAESAR SALAD', 'GREEN SALAD', 'TOSS SALAD', 'TOSSED GARDEN SALAD', 'CHEF SALAD', 'CHEF SALAD', 'SALAD', 'TOSSED SALAD', 'DARK MIXED GREENS', 'FIDDLEHEAD GREENS(FERNS)', 'LETTUCE', 'PARSLEY', 'ROMAINE SALAD', 'SPINACH', 'BABY MUSTARD GREEN', 'MEDITERRANEAN SALAD (ITALIAN DRESSING)', 'SAUTEED CHOI SUM', 'PUREED SAUTEED CHOI SUM'	
**MEAT_PROCESSED**	'SAUSAGE', 'BRATWURST', 'WIENER (FRANKFURTER)', 'SALAMI', 'PASTRAMI ON RYE', 'PASTRAMI ON RYE', 'LUNCHMEAT', 'KIELBASA', 'HOT DOG', 'BOLOGNA (BALONEY)', 'BEER SALAMI', 'BACON'	
**MILK_SOYMILK**	'ILK', 'MILK', 'DRINK_DAIRY', 'BEVERAGE'	"Beverage" was always some form of soymilk (flavoured/unflavoured)
**NUTS_SEEDS**	'NUTS', 'SEEDS'	
**PANCAKE**	'CHEESE BLINTZES', 'CREPE', 'WAFFLE', 'FRENCH TOAST', 'DUMPLING', 'DUMPLING', 'PUFF PASTRY'	
**PEANUT_BUTTER**	'PEANUT BUTTER', 'EANUT BUTTER'	
**PEAS**	'GREEN PEAS', 'PEAS', 'PEAS AND ONIONS', 'PEAS', 'PEAS_TV', 'PEA MINCE','PUREE GREEN PEAS', 'PUREE PEAS - SITE 30', 'PUREE SEASONED GREEN PEAS', 'PUREED PEAS', 'PUREED PEAS', 'SEASONED GREEN PEAS', 'SEASONED GREEN PEAS'	
**PIE**	'PIE', 'PIE CRUST', 'PIE FILLING', 'PUREE APPLE PIE', 'PUREE CHERRY PIE', 'PUREED APPLE PIE', 'DANISH PASTRY', 'PUMPKIN PIE MIX', 'PUREE CHERRY PIE', 'STRUDEL', 'TART', 'PUREED STRUDEL_PMH'	
**PORK**	'LONGE DE PORC AUX TOMATES', 'HAM'	
**POTATO**	'POTATO', 'MASH POTATO', 'MASHED POTATO', 'HASH BROWNS', 'HASHBROWN'	
**POULTRY**	'TURKEY', 'POULTRY FOOD PRODUCTS', 'NUGGETS', ' POULET CHINOIS À L''ANANAS', 'CHICKEN', 'BREAST', 'STRIPS'	
**PROTEIN_POWDER**	'PROTEIN', 'UNFLAVORED POWDER OF WHEY PROTEIN ISOLATE'	PROTEIN was always either beneprotein or whey

**PROTEIN_VEG**	'MAH PO TOFU', 'PUREED MAH PO TOFU', 'PUREE VEGETARIAN NUGGETS', 'VEGETARIAN MEAT', 'VEGETARIAN PATTY'	
**PUDDING**	'CREME CARAMEL', 'CREME CARAMEL_PMH', 'CUSTARD', 'PUDDING'	
**PUREE_SUPPER**	'PUREE STIR FRY TONG HO', 'PUREE SUPPER FRI WEEK 5', 'PUREE SUPPER FRI WEEK 6', 'PUREE SUPPER SAT WEEK 5', 'PUREE SUPPER SAT WEEK 6', 'PUREE SUPPER WED WEEK 5', 'PUREE SUPPER WED WEEK 6'	14 instances of PUREE_SUPPER
**SANDWICH**	'SANDWICH', 'SANDWICH', 'SANDWHICH', 'SANDWICH', 'GRILLED CHEESE', ' CHAUDRÉE DE MAIS', 'SLOPPY JOE', 'SLOPPY JOE', 'SLOPPY JOE', 'TUNA MELT', 'TUNA MELLT - SITE 30', 'TUNA MELLT - SITE 30*', 'TUNA MELT', 'TUNA MELT SN MB', 'TUNA MELT', 'TUNA MELLT - SITE 30 *'	
**SAUCE**	'DILL SAUCE_TV', 'DIP', 'GRAVY', 'CHEESE SAUCE', 'CREAM SAUCE SCR', 'MEAT SAUCE', 'ROSE SAUCE', 'RUSSIAN SALAD (RE)', 'SALAD DRESSING', 'SAUCE', 'SPANISH SAUCE'	
**SNACK**	'SNACK', 'SNACKS'	22 instances only ever charted as snack or snacks.
**SOUP_STEW**	'SOUP', 'BORSCHT', 'STEW', 'BROTH', 'CHILI', 'CHILLI', 'CHOWDER', 'SEAFOOD CONGEE', 'DRIED BOK CHOY & DRIED OYSTER CONGEE'	
**VEG_CRUC**	'BROCCOLI', 'CABBAGE', 'CAULIFLOWER', 'COLESLAW', 'KAI LAN', 'PUREE KAI LAN'	
**VEG_OTHER**	'ASPARAGUS', 'BRUSSEL SPROUT', 'BRUSSEL SPROUTS', 'BRUSSELS SPROUT', 'CELERY', 'CORN', 'MUSHROOM', 'OLIVES', 'ONION', 'PUREE SAUTEE SHANGHAI BOK CHOY', 'PUREE STIR FRIED BABY BOK CHOY', 'PUREED BABY MUSTARD GREEN', 'SAUTEE BABY BOK CHOY', 'SAUTEE SHANGHAI BOK CHOY', 'SAUTEE TAIWANESE BOK CHOY', 'SAUTEED MIXED PEPPERS', 'SAUTEED ONIONS', 'SUNRISE VEG', 'TURNIP', 'TRIO MIXED VEG (MINCED AND REGULAR) ', 'TRIO MIXED VEG (MINCED AND REGULAR)', 'VEGETABLE', 'VEGETABLES', 'WHITE FUNGUS', 'ZUCCHINI', 'BEET', 'CUCUMBER', 'FIDDLEHEAD GREENS (FERNS)', 'MARINATED MUSHROOM SALAD', 'PARSNIP', 'PEPPER', 'BOILED DINNER VEG', 'LO HAN CHAI', 'PUREE LO HAN CHAI', 'STIR FRY TONG HO'	Rhubarb was not included here because it was always cooked with sugar.
**WHOLE_GRAIN**	'COUSCOUS', 'CRANBERRY WALNUT QUINOA SALAD_TV', 'GRAINS', 'KASHA', 'QUINOA SALAD W/ CRANBERRIES', 'RICE'	

**YOGURT_DAIRY_OTHER**	'KEFIR', 'COTTAGE CHEESE', 'YOGURT', 'YOGOURT'
**BAR**	'Nanaimo Bar', 'Bar, dessert, lemon lover's', 'Bar, diet, high protein, granola, chocolate chip', 'GRANOLA BAR, CEREAL BAR, FRUIT-FILLED, ALL FLAVOURS','Pumpkin Bar'

Table S2 Resident sample demographics based on daily energy and protein by subgroup

	Variable N (%)	**Energy intake (kcal/day)**	**Protein intake (g/day)**
All Residents	634 (100)	1561.0 (418.0)	58.0 (18.3)
Sex			
Male	195 (30.7)	1728.2 (421.3)	65.5 (18.7)
Female	439 (69.2)	1486.8 (394.8)	54.7 (17.1)
Diet type			
Regular	327 (51.6)	1575.9 (405.3)	57.5 (17.5)
Minced meat	20 (3.2)	1571.5 (623.2)	58.3 (25.1)
Minced	84 (13.2)	1462.2 (401.5)	56.2 (19.0)
Pureed	63 (9.9)	1572.0 (401.3)	61.7 (21.1)
Other	140 (22.1)	1579.1 (426.8)	58.5 (17.4)
Nutritional status			
Malnourished	80 (12.6)	1376.7 (459.0)	52.3 (20.4)
At-risk	261 (41.2)	1501.3 (393.1)	56.4 (17.8)
Normal	292 (46.1)	1663.0 (400.9)	60.8 (17.6)

Table S3 Summary of ANOVA and Tukey HSD post-hoc results analysing DII scores by sex, texture, and nutritional status.

Variable	Source	SS	df	MS	F	p-value
Sex	Groups	20.955	1	20.9548	16.4	5.79E-05
	Error	807.416	632	1.2776		
	Total	828.37	633			
	Post-hoc with Tukey HSD: p-value (μ [CI])					
	Male vs. Female: 0.000051 (-0.393946 [-0.584593, -0.203300])					
Texture	Groups	13.398	4	3.34961	2.59	0.0361
	Error	814.971	629	1.29566		
	Total	828.37	633			
	Post-hoc with Tukey HSD: p-value (μ [CI])					
	Minced vs. Pureed: 0.022931 (0.568399 [0.050907, 1.085890])					
Nutritional Status	Groups	6.568	2	3.28384	2.55	0.0791
	Error	812.131	630	1.2891		
	Total	818.698	632			
	Post-hoc with Tukey HSD: p-value (μ [CI])					
	Malnourished vs. Normal: 0.075789 (0.311395 [-0.024404, 0.647194])					

SS: sum of squares; df: degrees of freedom; MS: mean square; F: F-statistic of variation between sample means over the variation within samples; CI: confidence interval; μ: mean difference between groups identified
